# Chronic colitis exacerbates NLRP3-dependent neuroinflammation and cognitive impairment in middle-aged brain

**DOI:** 10.1186/s12974-021-02199-8

**Published:** 2021-07-06

**Authors:** Xiao-fei He, Li-li Li, Wen-biao Xian, Ming-yue Li, Li-ying Zhang, Jing-hui Xu, Zhong Pei, Hai-qing Zheng, Xi-quan Hu

**Affiliations:** 1 Department of Rehabilitation Medicine, The Third Affiliated Hospital, Sun Yat-sen University, 600 Tianhe Road, Guangzhou, 510630 Guangdong China; 2 Department of Neurology, National Key Clinical Department and Key Discipline of Neurology, Guangdong Key Laboratory for Diagnosis and Treatment of Major Neurological diseases, The First Affiliated Hospital, Sun Yat-sen University, Guangzhou, 510080 Guangdong China

**Keywords:** Inflammatory bowel disease, Cognition, Glymphatic clearance, NLRP3 inflammasome, T cell

## Abstract

**Background:**

Neuroinflammation is a major driver of age-related brain degeneration and concomitant functional impairment. In patients with Alzheimer’s disease, the most common form of age-related dementia, factors that enhance neuroinflammation may exacerbate disease progression, in part by impairing the glymphatic system responsible for clearance of pathogenic beta-amyloid. Inflammatory bowel diseases (IBDs) induce neuroinflammation and exacerbate cognitive impairment in the elderly. The NACHT-LRR and pyrin (PYD) domain-containing protein 3 (NLRP3) inflammasome has been implicated in neuroinflammation. Therefore, we examined if the NLRP3 inflammasome contributes to glymphatic dysfunction and cognitive impairment in an aging mouse model of IBD.

**Methods:**

Sixteen-month-old C57BL/6J and NLRP3 *knockout* (KO) mice received 1% wt/vol dextran sodium sulfate (DSS) in drinking water to model IBD. Colitis induction was confirmed by histopathology. Exploratory behavior was examined in the open field, associative memory by the novel-object recognition and Morris water maze tests, glymphatic clearance by in vivo two-photon imaging, and neuroinflammation by immunofluorescence and western blotting detection of inflammatory markers.

**Results:**

Administration of DSS induced colitis, impaired spatial and recognition memory, activated microglia, and increased A1-like astrocyte numbers. In addition, DSS treatment impaired glymphatic clearance, aggravated amyloid plaque accumulation, and induced neuronal loss in the cortex and hippocampus. These neurodegenerative responses were associated with increased NLRP3 inflammasome expression and accumulation of gut-derived T lymphocytes along meningeal lymphatic vessels. Conversely, NLRP3 depletion protected against cognitive dysfunction, neuroinflammation, and neurological damage induced by DSS.

**Conclusions:**

Colitis can exacerbate age-related neuropathology, while suppression of NLRP3 inflammasome activity may protect against these deleterious effects of colitis.

**Supplementary Information:**

The online version contains supplementary material available at 10.1186/s12974-021-02199-8.

## Introduction

Neuroinflammation is strongly implicated in the pathogenesis of age-related cognitive decline, including that associated with Alzheimer’s disease (AD), the most common form of dementia in older adults [1]. Patients with AD show progressive accumulation of misfolded amyloid-beta (Aβ) protein within plaques, and plaque load is directly associated with the severity of neurodegeneration and eventual functional deficits. However, some plaques may be observed 10–20 years before the onset of cognitive decline [2], so there is a substantial therapeutic window for curtailing disease progression Indeed, numerous strategies have been examined to reduce Aβ accumulation and prevent or slow down AD progression [2], but there are currently no widely effective treatments. Growing evidence indicates that the accumulation of misfolded Aβ results from an imbalance between production and clearance [3], and that impairment of Aβ clearance is responsible for the most common type of AD [4] Thus, treatments that enhance Aβ clearance may be among the most broadly effective treatment strategies for AD.

The “glymphatic” pathway allows the exchange of para-arterial cerebrospinal fluid (CSF) with interstitial fluid (ISF) in the brain parenchyma, thereby promoting clearance of various toxic waste products from the central nervous system (CNS), including amyloid beta [5]. This pathway is markedly disrupted by neuroinflammation in aged brain [6, 7], which may lead to amyloid beta accumulation and concomitant neural damage. Glymphatic clearance is dependent on aquaporin 4 (AQP4) channels expressed at high density on the astrocytic endfeet abutting cerebral capillaries [8], but reactive astrogliosis in response to inflammatory signaling reduces AQP4 polarization [9]. Further, reactive astrocytes of the “A1” phenotype strongly expressing classical complement cascade genes are dramatically upregulated in the aged brain, contributing to neuronal death in many age-associated neurodegenerative diseases [10, 11]. Thus, age-related neuroinflammation may impair glymphatic function.

There is also growing evidence for a regulatory role of the gut–brain axis in neuroinflammation and cognition. For example, patients with inflammatory bowel diseases (IBDs) show an elevated incidence of cognitive impairment compared to age-matched individuals without IBDs [12]. Moreover, prevention of bowel inflammation by germ-free rearing and antibiotic treatment reduces cerebral Aβ pathology and neuroinflammation in AD model mice [13, 14]. However, it is unclear how astrocytic function and glymphatic clearance are influenced by the gut–brain axis in the elderly.

The NACHT, LRR, and pyrin (PYD) domain-containing protein 3 (NLRP3) inflammasome is implicated in both gut immune homeostasis and neuroinflammation [15], and activation was found to exacerbate Aβ deposition and cognitive impairment in AD [16]. Dextran sodium sulfate (DSS)-induced colitis is the most widely used experimental animal model of IBD due to the resemblance of this condition with human IBD [17]. Ingestion of DSS induces intestinal inflammation by directly damaging the outer monolayer of colon epithelial cells, allowing intestinal contents to cross into the underlying tissue [18]. In the present study, we investigated possible contributions of the NLRP3 inflammasome to exacerbation of neurological dysfunction by DSS-induced colitis in aging mice. We found that oral administration of DSS to wild-type mice for 4 weeks increased NLRP3 inflammasome activity and gut-derived T cell numbers along meningeal lymphatic vessels (mLVs), induced microglial and astrocyte activation, impaired glymphatic clearance of Aβ, and aggravated cognitive decline. In contrast, these responses were not found in NLRP3 *knockout* (KO) mice. Collectively, these results identify the NLRP3 inflammasome as a potential therapeutic target for AD and other neuroinflammatory disorders exacerbated by colitis.

## Materials and methods

### Animals

The study was approved by the Animal Research Committee of Laboratory Animal Monitoring Institute of Guangdong Province (Guangzhou, China; committee’s reference number: [2013]97). All efforts were made to minimize the number and suffering. Both male and female mice were used in our study; NLPR3 KO mice were obtained from the Jackson laboratory (B6.129S6-Nlrp3tm1Bhk/J, Catalog number: 021302) and bred in the Laboratory Animal Monitoring Institute of Guangdong Province, they were founded on a C57BL/6J background. Wild-type (WT) C57BL/6J mice were provided from the Laboratory Animal Monitoring Institute of Guangdong Province. Male animals were used at sixteen months of age and were housed under a 12:12 h light: dark cycle (light on from 07:00 to 19:00 h), with controlled temperature and humidity. WT and NLRP3 KO mice were randomly divided into two groups: Control (Ctrl) and DSS. In the control group, mice received distilled water without DSS for 28 days. According to the modified procedure as described previously [19, 20], mice in the DSS group were treated with multiple-cycle administration of 1% wt/dextran sodium sulfate (DSS, molecular weight 30,000 to 50,000 g/mol, MP Biomedicals, CANADA) in drinking water on days 1 to 5, 8 to 12, 15 to 19, and 22 to 26, which was replaced by drinking water with fresh DSS solutions on day 6 to 7, 13 to 14, 20 to 21, and 27 to 28.

### Open field

Anxiety and exploratory activity were examined in the open field test [21]. The testing apparatus was a 50 × 50 cm square arena bounded by 40-cm walls. A video camera suspended above recorded spontaneous motor activity over 5-min trials. Mice (n = 12 per group, six male and six female) were placed in the center of the arena, and both total distance traveled and time spent in the center (Region of interest, ROI) were recorded as indices of exploratory activity and anxiety, respectively.

### Morris water maze task

**Fig. 1:**
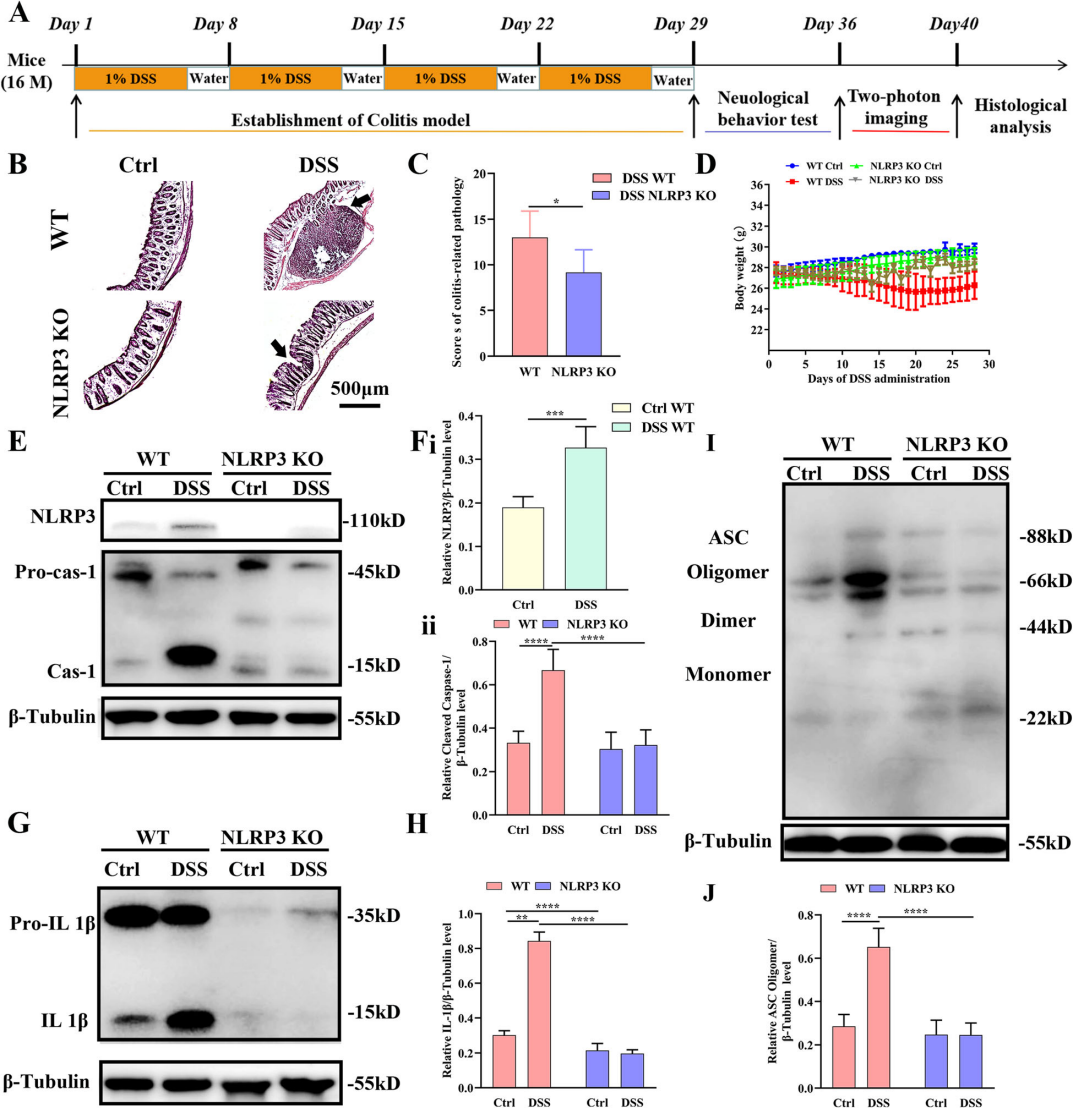
Addition of dextran sodium sulfate (DSS) to drinking water (1% vol/vol) for 4 weeks induced colitis and increased NLRP3 activation in the brain of WT mice. **A** Schematic diagram of the experimental design. **B** Hematoxylin and eosin (H&E) staining showing more severe damage in the colon of DSS-fed WT mice compared to DSS-fed NLRP3 KO mice. **C** Comparison of colitis-related pathology scores among WT, DSS-fed WT, NLRP3 KO, and DSS-fed NLRP3 KO mice. **D** Line diagram showing changes in DSS-fed WT mouse body weight during the establishment of colitis. **E** Chemiluminescence imaging of western blots showing that DSS feeding increased NLRP3 and caspase-1 expression levels in the brains of WT mice compared to untreated control (Ctrl) WT mice but not in NLRP3 KO mouse brain. **F** Comparisons of NLRP3/β-tubulin (i) and cleaved caspase 1/β-tubulin (ii) ratios among control WT, DSS-fed WT, control NLRP3 KO, and DSS-fed NLRP3 KO mice. **G** Chemiluminescence images of western blots showing increased brain expression of IL-1β by DSS-fed WT mice compared to Ctrl WT mice but not DSS-fed NLRP3 KO mice. **H** Comparisons of IL-1β/β-tubulin ratio among treatment groups. **I** Chemiluminescence image of ASC and β-tubulin immunoexpression by western blot showing that DSS feeding increased ASC oligomer expression in the brains of WT mice but not NLRP3 KO mice. **J** Comparisons of ASC oligomer/β-tubulin ratio among treatment groups. Each dataset is expressed as mean ± SD. *P ≤ 0.05; **P ≤ 0.01; ***P ≤ 0.001; ****P ≤ 0.0001. n = 6 mice

Water maze tasks were performed after the finish of the DSS administration as described previously (Fig. 1A) [22, 23]. Briefly, mice (n = 12 per group, six male and six female) received four trials (up to 60 s) on five consecutive training days, and then received a single 60-s probe trial on day 6. The latency to reach the platform during training days, the times crossing the target area (former platform position), and the time spent in the target quadrant during the probe trial were recorded.

### Novel object recognition memory test

Recognition memory was evaluated using the novel object test as described previously [24–26]. Mice were placed in the open field arena described above and allowed to acclimate for 1 h. Two 1-ml syringe barrels (Becton, Dickinson and Company, USA) were placed in the arena equidistant from the center, and the mouse was allowed to freely explore the environment for 10 min. Object exploration times, defined as nasal or oral contact durations, were recorded. Twenty-four hours later, mice were returned to the open field with one of the syringes replaced by a bottle cap (novel object), and contact durations with the now-familiar and novel objects recorded for 10 min. The difference in time spent exploring the novel object versus the familiar object was calculated as an index of novel object recognition.

### Detection of lymphocytes migrated from the gut to the meninges

After DSS administration, the fluorescent membrane dye CM-Dil (5 μM in 2 μL of PBS per PP) (Life Technologies, USA) was injected into Peyer’s patches (PPs) surrounding the ileum [19, 27]. After two-photon imaging, mice (n = 6 per group, three male and three female) were sacrificed and the meninges isolated for immunohistological analysis of CM-DiI labeling as described previously [28]. Briefly, mandibles and the skull rostral to the maxillae were removed, and the top of the skull with the meninges was collected and fixed in 4% paraformaldehyde (PFA) for 24 h at 4 °C. The meninges were dissected away from the skullcap, simultaneously permeabilized with 0.3% Triton X-100 and blocked with 10% goat serum for 1 h at room temperature, and then incubated overnight at 4 °C with primary rat anti-NLRP3 (1:100, Thermo Fisher, USA), rabbit anti-CD3 (1:100, Abcam, USA), and rabbit anti-LYVE-1 (1:100, Abcam, USA). Immunolabeled tissues were then incubated with an Alexa Fluor® 555-conjugated anti-rabbit IgG [(H+L), F(ab')2 Fragment (1:300, Cell Signaling Technology)] and Alexa Fluor® 488-conjugated anti-rat IgG [(H+L) (1:300, Cell Signaling Technology, USA)] in PBS containing 10% normal goat serum at room temperature for 1 h. Fluorescence images were acquired using a confocal microscope (Leica, Germany).

### In vivo two-photon imaging of glymphatic clearance

The efficiency of glymphatic clearance was evaluated using in vivo two-photon imaging [7]. Briefly, mice (n = 6 per group, 3 male and 3 female) were anesthetized and a thin cranial window was created at the parietal. Fluorescein isothiocyanate (FITC)-dextran (70 kDa; Sigma-Aldrich, USA) was dissolved in artificial cerebrospinal fluid at a concentration of 1%; 10 μl of FITC was injected into the cisterna magna using a microsyringe connected with a syringe pump controller. 0.2 ml of 1% rhodamine B (Sigma-Aldrich, USA) in saline was injected intravenously to show the brain vascular before imaging. Two-photon imaging on the right parietal cortex (2 mm caudal from bregma, and 1.7 mm lateral from the midline) was performed using a two-photon laser scanning microscope (Leica, Germany) equipped with a water immersion objective (25×). To monitor the clearance of FITC-dextran injected into the brain parenchyma, three-dimensional (3D) xyz stacks (512 × 512 pixels, 2-μm resolution) were taken up to 300 μm below the cortical surface at 5, 15, 30, 45, and 60 min after the injection of the FITC-dextran, the overall fluorescence intensities were analyzed. Besides, images 100 μm below the cortical surface were obtained and the fluorescence intensities in the paravascular space were analyzed to examine the efficiency of glymphatic clearance.

### Histology

Mice (n=6 per group, three male and three female mice) were perfused with 50 ml ice-cold phosphate buffer saline (PBS) and 50 ml of 4% (w/v) paraformaldehyde in PBS. For immunofluorescence staining, coronal brain slices of right parietal cortex with 10 μm thick at interval of 100 μm or consecutive 40 μm were sectioned. Brain sections were boiled in citric acid buffer for 5 min in a microwave oven and were treated with 0.3% Triton X-100 and 10% goat serum for 1 h at room temperature and then incubated overnight at 4 °C with primary antibody [1:300 rabbit anti-ionized calcium binding adapter molecule 1 (Iba1) antibody, Wako, Japan; 1:100 rat anti-NLRP3 antibody, Thermo Fisher, USA; 1:100 mouse purified anti-β-amyloid, 1-42 antibody, BioLegend, USA; 1:100 mouse purified anti-β-amyloid, 1-40 antibody, BioLegend, USA; 1:300 rabbit anti-MAP2 antibody, Affility Biosciences, USA; 1:300 mouse anti-GFAP, Sigma-Aldrich, USA; 1:100 rabbit anti-Complement C3 antibody, Thermo Fisher, USA; 1:200 rabbit anti-AQP4, Peptide, USA; 1:300 anti-mouse NeuN, Millipore, USA] and then incubated with a secondary antibody [1:300 Anti-mouse IgG (H+L), F(ab')_2_ Fragment (Alexa Fluor® 488 Conjugate), Cell signaling technology, USA; 1:300 Anti-rabbit IgG (H+L), F(ab')2 Fragment (Alexa Fluor® 555 Conjugate, Cell signaling technology, USA; 1:300 Anti-rat IgG (H+L), (Alexa Fluor® 555 Conjugate), Cell signaling technology, USA] in PBS containing 10% normal goat serum at room temperature for 1 h. Images for Aβ were acquired using a Nikon fluorescence microscope (Nikon, Japan), images for other histological analysis were acquired using a confocal microscope (Leica, Germany).

### Grading of intestinal inflammation-related dysfunction

Body weight was monitored daily during DSS administration, and induction of colitis was confirmed by hematoxylin and eosin (H&E) staining and histological scoring. Briefly, 2-cm pieces of the distal colon were isolated, washed in cold phosphate-buffered saline (PBS), and cut into 10-μm thick sections at 100-μm intervals using a freezing microtome (Leica, Germany). Sections were stained with H&E, and colitis-related pathology graded as follows according to a previous study with modifications [29–32]: body weight loss (0, none; 1, 1−5%; 2, 5−10%; 3, 11−15%; 4, > 15%) plus diarrhea severity (0, normal; 2, loose stools; 4, watery diarrhea) plus anal bleeding severity (0, normal; 2, slight bleeding; 4, gross bleeding) plus severity of inflammation (0 none, 1 mild, 2 moderate, 3 severe) plus extent of inflammation (0 none, 1 mucosa, 2 mucosa and submucosa, 3 transmural) plus crypt damage (0 none, 1 1/3 damaged, 2 2/3 damaged, 3 crypts lost, surface epithelium present, 4 crypts and surface epithelium lost). Adding the score for each parameter yielded a maximum score of 22.

### Western blotting

Six mice (three male and three female) in each group were perfused with 50 mL ice-cold PBS. Tissues from whole cortex and hippocampus were homogenized in 500 μL 1× lysis buffer in a Precellys homogenizer (Stretton Scientific, Derbyshire, UK) and total protein levels were quantified using a Pierce™ Microplate BCA Protein Assay Kit (Thermo Fisher Scientific, USA) according to the manufacturer’s instructions. Proteins were separated at 30 μg per gel lane by sodium dodecyl sulfate polyacrylamide gel electrophoresis (SDS-PAGE) at 200 V for 45 min using 4%–12% precast polyacrylamide gels (Novex, Invitrogen). Separated proteins were transferred to polyvinylidene fluoride membranes (Millipore, Bedford, MA, USA) at 120 V for 1.5 h. Membranes were blocked in 5% fat-free skim milk power (R&D Systems, Minneapolis, MN, USA) for 1 h and incubated with the following primary antibodies overnight at 4 °C: mouse anti-NLRP3 (Thermo Fisher, USA), rabbit anti-IL-1beta (Abcam, USA) rabbit anti-caspase-1 (Abcam, USA) rabbit anti-apoptosis-associated speck (ASC, Affinity Biosciences, USA), rabbit anti-tubulin beta (Affinity Biosciences, USA), rabbit anti-beta amyloid 1-40 antibody (Abcam, USA), rabbit anti-beta amyloid 1-42 antibody (Abcam, USA), and rabbit anti-APP (Cell Signaling Technology, USA). Membranes were then incubated with secondary antibody (anti-mouse IgG, HRP-linked Antibody or anti-rabbit IgG, HRP-linked Antibody, Both from Cell Signaling Technology, USA) for 1 h in a dark room. Target protein bands were visualized and quantified using a chemiluminescence imaging system.

### Statistical analyses

The 3D image overlays were visualized and analyzed with the Leica Application Suite (LAS) Advanced Fluorescence Lite software (LAS AF Lite, 2.4.1 build 6384, Leica, Germany). The ImageJ software (National Institutes of Health, Bethesda, MD, USA) was used to analyze the histological and western blotting results. For histological scoring of colon and NLRP3 expression, independent-samples t test was used to analyze. For other data*,* two-way repeated measures ANOVA with further turkey’s multiple tests were used to analyze. A *P* value *<*0.05 was considered statistically significant (Prism 8.0, GraphPad software, La Jolla, CA, USA). Data are expressed as means ± standard deviations of the means (SD).

## Results

### DSS administration induced colitis and activated the NLRP3 inflammasome in the brain

We first compared histological signs of colitis, brain expression levels of the NLRP3 inflammasome, and accumulation of downstream pro-inflammatory factors between WT and NLRP3 KO mice under control conditions and following oral DSS administration for 4 weeks. Treatment groups (control and DSS-treated WT and NLRP3 KO mice) were then subjected to a battery of additional immunohistological and behavioral analyses (Fig. 1A). First, we confirmed that DSS administration induced colitis in WT mice by H&E staining (Fig. 1B). Histological score was significantly lower in DDS-treated NLRP3 KO mice than WT mice (t = 2.46, *P* < 0.05) (Fig. 1C), indicating that colitis was NLRP3-dependent. Furthermore, body weight was significantly reduced in DSS-treated WT mice but increased progressively in the other groups (Fig. 1D).

Oral DSS significantly enhanced expression of the NLRP3 inflammasome in wild-type (WT) mouse brain as evidenced by western blotting (Fig. 1E), while no NLRP3 expression was detectable in NLRP3 KO mouse brain following DSS administration (*P* < 0.001 vs. WTs) (Fig. 1E and F i). Dual-immunofluorescence staining revealed NLRP3 inflammasomes in microglia (Supplementary figure 1A) and astrocytes (Supplementary figure 1. B) but not in neurons (Supplementary figure 1C). Furthermore, DSS administration increased cleaved caspase-1 expression in the brain of WT mice (*P* < 0.0001) but not NLRP3 KO mice (*P* > 0.05), and post-DSS expression of cleaved caspase-1 was significantly lower in DSS-treated NLRP3 KO mice than DSS-treated WT mice (*P* < 0.0001) (Fig. 1E and F ii).

The NLRP3 inflammasome drove the inflammatory response in part by cleaving immature interleukin (IL)-1β to yield the active form. In WT mice, brain expression of IL-1β was significantly greater following DSS treatment compared to WT controls (*P* < 0.0001), while no such change was detected in NLRP3 KO mice (*P* > 0.05), and expression was significantly lower in NLRP3 KO mice compared to WT mice following DSS (*P* < 0.0001) (Fig. 1G and H). Similarly, expression of the ASC oligomer was significantly greater in DSS-treated WT mice compared to control WT mice (*P* < 0.0001), while there was no difference in expression between control and DSS-administered NLRP3 KO mice (*P* > 0.05), and expression was significantly lower in NLRP3 KO mice compared to WT mice following DSS (*P* < 0.0001) (Fig. 1I and J). These results indicated that DSS administration activated the NLRP3 inflammasome in WT mice and increased the expression of the pro-inflammatory cytokine IL-1β.

### NLRP3 depletion protected against colitis-induced neurological dysfunction

**Fig. 2:**
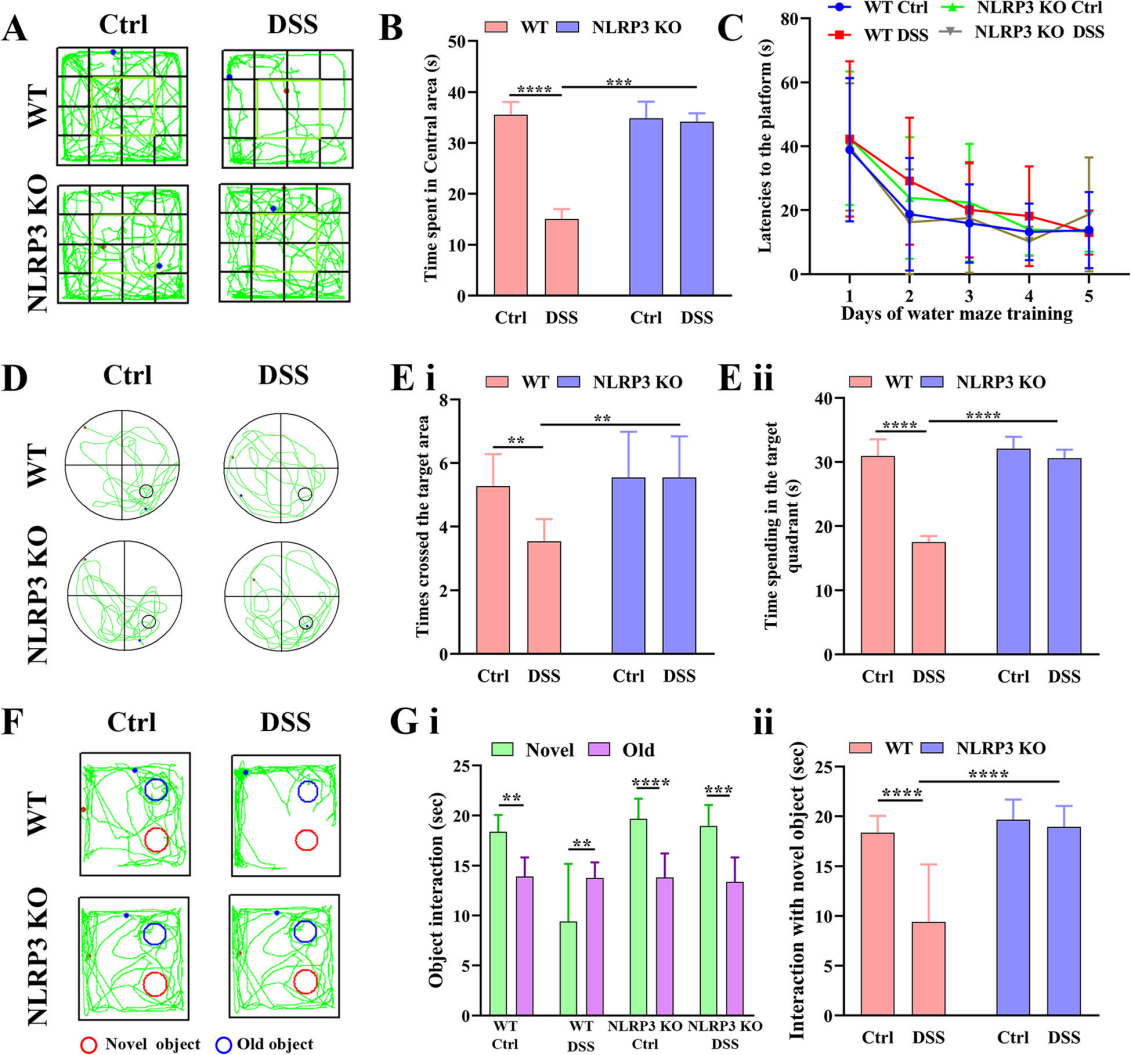
Induction of colitis by addition of DSS to drinking water (1% vol/vol) for 4 weeks altered spontaneous motor behavior and impaired spatial and recognition memory in aged (16-month-old) wild-type (WT) mice but not age-matched NLRP3 KO mice. **A** Representative movement tracks in the open field test showing less time spent in the central area by DSS-fed WT mice compared to untreated control (Ctrl) WT mice but no difference between DSS-fed and control NLRP3 KO mice. **B** Comparison of time spent in the central area of the open field by all 4 treatment groups. **C** Comparison of latency to the platform during the 5 days of Morris water maze training. **D** Representative swim paths during the probe trial for spatial memory showing that DSS-fed WT mice made fewer crossings over the former platform location and spent less swim time in the target quadrant than control WT mice, indicating spatial memory impairment, while these values did not differ between DSS-fed and control NLRP3 KO mice. **E** Comparison of times crossing the former target area (i) and time spent in the target quadrant in the probe trial (ii). **F** Representative movement tracks in the novel object test showing that DSS-fed WT mice spent equal time contacting the familiar and novel objects, while mice in other treatment groups spent more time in contact with the novel object. **G** Comparison of the time spent in contact with the novel and familiar objects by all 4 treatment groups (i) and comparison of the time spent in contact with the novel object among the four groups (ii). Each dataset is expressed as mean ± SD. *P ≤ 0.05; **P ≤ 0.01; ***P ≤ 0.001; ****P ≤ 0.0001. n = 12 mice

Control and DSS-fed WT and NLRP3 KO mice were then compared for spontaneous behaviors in the open field and for cognitive functions using the Morris water maze and novel object recognition tasks. Time spent in the center of the open field (Fig. 2A), a behavioral index of anxiety, differed significantly among groups, and pair-wise comparisons revealed significantly lower center time in the DSS-treated WT group, suggesting greater anxiety, compared to control WT mice (*P* < 0.0001). In contrast, there was no difference in center time between DSS-administered and control NLRP3 KO mice (*P* > 0.05). Moreover, DSS-fed NLPR3 KO mice spent more time in the central area compared to DSS-fed WT mice (*P* < 0.001) (Fig. 2B). These increases in anxiety appear to depend on induction of colitis rather than DSS ingestion per se.

Morris water maze performance also indicated significant cognitive dysfunction in DSS-fed WT mice but not in NLPR3 KO mice. During the training phase to find a hidden platform (Fig. 2C), there were no group differences within training days (all *P* > 0.05). However, in the probe trail for spatial memory in which the hidden platform was removed, administration of DSS significantly reduced the number of former platform crossings among DDS-fed WT mice (*P* < 0.01 vs. WT controls) but not NLRP3 KO mice (*P* > 0.05 vs. control KO mice), and the number of former platform location crossing was significantly greater among DSS-fed NLRP3 KO mice than DSS-fed WT mice (*P* < 0.01) (Fig. 2D and E i).Similarly, administration of DSS significantly reduced target quadrant time among WT mice compared to control WT mice (*P* < 0.0001) but had no effect on the performance of NLRP3 KO mice (*P* > 0.05), and DSS-fed NKRP3 KO mice spent more time in the target quadrant than DSS-fed WT mice *(P* < 0.0001) (Fig. 2D and E ii). Collectively, these findings suggest that colitis induces a NLRP3 inflammasome-dependent spatial memory deficit.

Further, recognition memory was also impaired in DSS-fed WT mice but not DSS-fed NLRP3 KO mice as evidenced by the novel object recognition test [25]. As expected, control WT, control NLRP3 KO, and DSS NLRP3 KO groups spent more time in contact with the novel object than the previously presented (familiar) object (*P* < 0.01, *P* < 0.0001, and *P* < 0.001, respectively) (Fig. 2F and G i). However, DSS-fed WT mice spent less time in contact with the novel object than the familiar object (*P* < 0.01), while time spent in contact with the familiar object did not differ significantly among the other treatment groups (all *P* > 0.05). Treatment of WT mice with DSS significantly reduced the time contacting the novel object (*P* < 0.0001) but DSS had no effect on NLRP3 KO mice (*P* > 0.05), and time contacting the novel object was significant higher among DSS-fed NLRP3 KO mice than DSS-fed WT mice (*P* < 0.0001) (Fig. 2F and G).

### NLRP3 depletion inhibited colitis-induced microglial activation and protected against neuronal loss

**Fig. 3:**
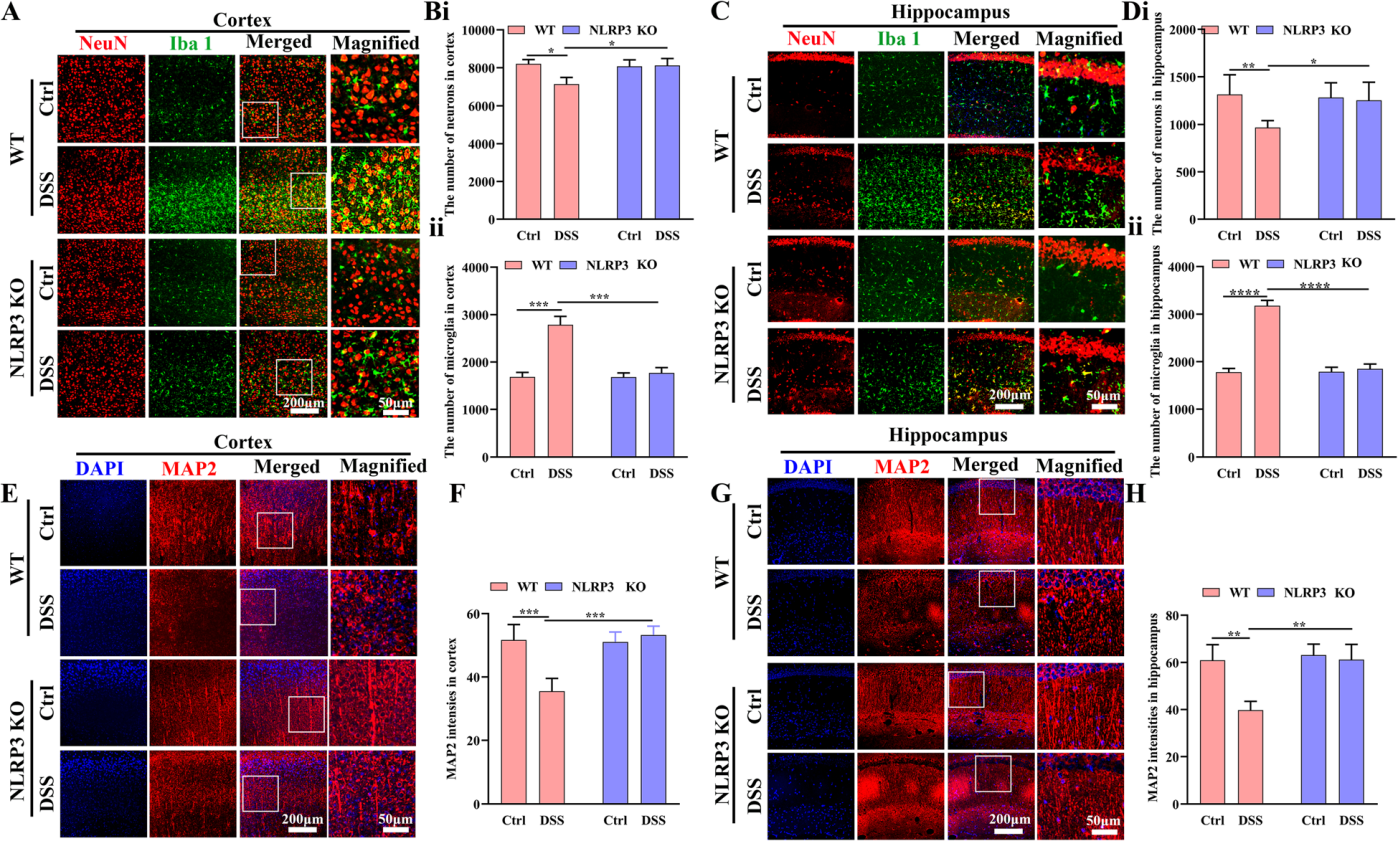
NLPR3 *knockout* protected against neuroinflammation and neurodegeneration from DSS administration. **A** Immunofluorescence staining of neurons and microglia in the cortex (25× water immersion objective). **B** Comparison of neuronal (i) and microglia (ii) numbers among control WT, DSS-fed WT, NLRP3 KO, and DSS-fed NLRP3 KO mice (average of four fields/slice, five slices per mouse, 6 mice per group). **C** Immunofluorescence staining of neurons and microglia in the hippocampus (CA1 area, 25× water immersion objective). **D** Comparison of neuronal (i) and microglial (ii) number among treatment groups (CA1 area, average of two fields/slice, five slices per mouse, 6 mice per group). **E** Immunofluorescence staining of MAP2 (25× water immersion objective) in the cortex. **F** Comparison of MAP2 immunostaining intensity in the cortex among treatment groups (average of four fields/slice, five slices per mouse, 6 mice per group). **G** Immunofluorescence staining of MAP2 in the hippocampus (CA1 area, 25× water immersion objective). **H** Comparison of MAP2 staining intensity in the hippocampus among treatment groups (average four fields/slice, five slices per mouse, 6 mice per group). Each dataset is expressed as mean ± SD. *P ≤ 0.05; **P ≤ 0.01; ***P ≤ 0.001; ****P ≤ 0.0001. n = 6 mice

We then analyzed the effect of DSS on neuronal survival and the potential protection conferred by NLRP3 depletion. Consistent with the cognitive dysfunction induced by DSS in WT but not NLRP3 mice, cortical neuron number was significantly lower in DSS-fed WT mice than control WT mice (*P* < 0.05) while there was no difference between DSS-fed and control NLRP3 KO mice (*P* > 0.05). The number of cortical neurons was also greater in DSS-fed NLRP3 KO mice than DSS-fed WT group (*P* < 0.05) (Fig. 3A and Bi). In contrast, the number of cortical microglia was significantly higher in DSS-fed WT mice compared to control WT mice (*P* < 0.001) while there was no difference between DSS-fed and control NLRP3 KO mice (*P* > 0.05). The number of cortical microglia was also lower in DSS-fed NLRP3 KO mice than DSS-fed WT mice (*P* < 0.001) (Fig. 3A and B ii), suggesting the suppression of NLRP3 inflammasome activity protected against colitis-induced neuroinflammation and neurodegeneration. In the hippocampus as well, hippocampal neuron number was significantly lower in DSS-fed WT mice than control WT mice (*P* < 0.01) but did not differ between DSS-fed and control NLRP3 KO mice (*P* > 0.05), and hippocampal neuron number was significantly higher in DSS-fed NLRP3 KO mice than DSS-fed WT mice (*P* < 0.05) (Fig. 3C and D i). Similar to the cortex, the number of microglia was significantly higher in the hippocampus of DSS-fed WT mice compared to control WT mice (*P* < 0.0001) but did not differ between DSS-fed and control NLRP3 KO mice (*P* > 0.05) and was significantly lower in DSS-fed NLRP3 KO mice than DSS-fed WT group (*P* < 0.0001) (Fig. 3C and D ii). These results indicated that DSS administration induced neuronal loss in the hippocampus and that this neurodegenerative response was dependent on NLRP3.

We also quantified neuronal functional integrity by immunostaining for MAP2 in the cortex and hippocampus. Mean MAP2 expression intensity was lower in the cortex of DSS-fed WT mice than control WT mice (*P* < 0.001), but did not differ between control and DSS-fed NLRP3 KO mice (*P >* 0.05). Cortical MAP2 expression was also significantly higher in DSS-fed NLRP3 KO mice than DSS-fed WT mice (*P* < 0.001) (Fig. 3E and F). In the hippocampus as well (Fig. 3G and H), MAP2 expression intensity was significantly lower in DSS-fed WT mice than control WT mice (*P* < 0.01) but did not differ between DSS-fed and control NLRP3 KO mice (*P* > 0.05) was significant greater in DSS-fed NLRP3 KO mice than DSS-fed WT mice (*P* < 0.01).

### NLRP3 depletion attenuated the colitis-induced amyloid beta deposition

**Fig. 4:**
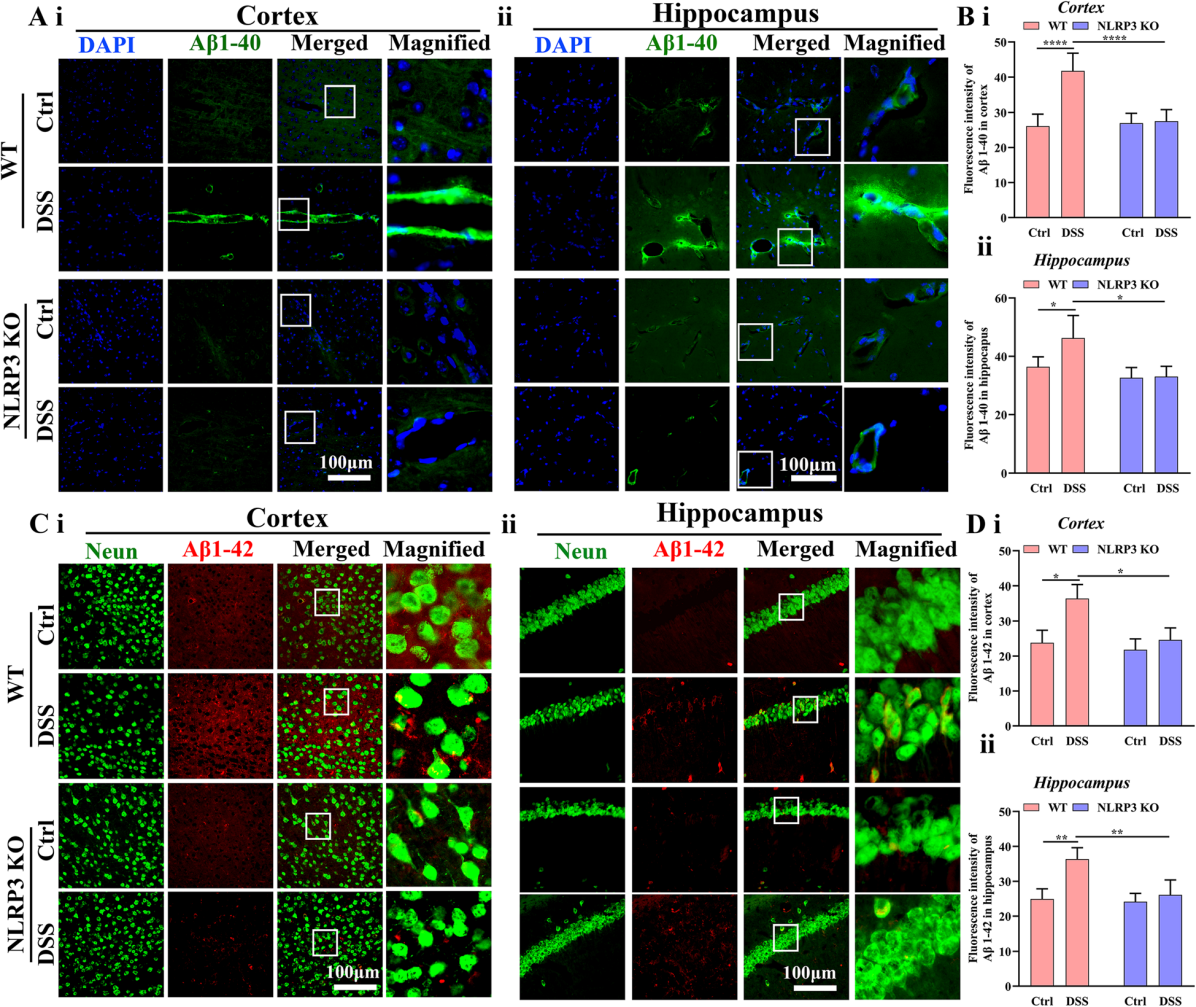
Colitis increased Aβ accumulation in WT mice but not NLRP3 KO mice. **A** Representative images of Aβ1–40 fragment immunoexpression in the cortex (i) and hippocampus (ii) (Left 3 rows: 40× objective; right row: expanded images (3×) of regions in the white boxes). **B** Comparison of Aβ1–40 expression intensity in the cortex (i) and hippocampus (ii) among control WT, DSS-fed WT, NLRP3 KO, and DSS-fed NLRP3 KO mice. **C** Representative images of Aβ1–42 fragment immunoexpression and neurons in the cortex (i) and hippocampus (ii) (Left 3 rows: 40× objective; right row: expanded images (3×) of regions in the white boxes). **D** Comparison of Aβ1–42 expression intensity in the cortex and hippocampus among treatment groups. Each dataset is expressed as mean ± SD. *P ≤ 0.05; **P ≤ 0.01; ***P ≤ 0.001; ****P ≤ 0.0001. n = 6 mice

Among individuals destined to develop age-related mild cognitive impairment (MCI) and AD, neuronal loss and neuroinflammation is associated with accumulation of Aβ, so we analyzed the effect of DSS administration on deposition of pathogenic Aβ (Aβ1–40, Aβ1–42) and the potential protective efficacy of NLRP3 depletion (Fig. 4). Consistent with cognitive and histological evaluations, administration of DSS significantly increased Aβ1–40 expression in the cortex of WT mice (*P* < 0.0001) but not NLRP3 KO mice (*P* > 0.05), and cortical Aβ1–40 expression was significantly lower in DSS-fed NLRP3 KO mice then DSS-fed WT mice (*P* < 0.0001) (Fig. 4A i and B i). In the hippocampus as well (Fig. 4A ii and B ii), DSS administration significantly increased Aβ1–40 deposition in WT mice (*P* < 0.05) but not NLRP3 KO mice (*P* > 0.05), and Aβ1–40 expression was significantly lower in DSS-fed NLRP3 KO mice than DSS-fed WT mice (*P* < 0.05). Similarly, administration of DSS also significantly increased Aβ1–42 deposition in the cortex of WT mice (*P* < 0.05) but not NLRP3 KO mice (*P* > 0.05), and Aβ1–42 expression was significantly lower in DSS-fed NLRP3 KO mice than DSS-fed WT mice (*P* < 0.05) (Fig. 4C i and D i), DSS administration significantly increased hippocampal Aβ1–42 deposition in WT mice (*P* < 0.01) but not NLRP3 KO mice (*P* > 0.05), while Aβ1–42 intensity was significantly lower in DSS-fed NLRP3 KO mice than DSS-fed WT mice (*P* < 0.05) (Fig. 4C ii and D ii). We also measured amyloid precursor protein (APP) and Aβ fragments (Supplementary Fig. 2) by western blotting. There were no significant pair-wise differences in APP expression levels among the four treatment groups (all *P* > 0.05) (Supplementary Fig. 2 A and B i), while both Aβ1–40 (Supplementary Fig. 2 A and B ii) and Aβ1–42 (Supplementary Fig. 2 C and D) expression levels were higher in DSS-fed WT mice than control WT mice (*P* < 0.0001, *P* < 0.05) but did not differ between DSS-fed and control NLRP3 KO mice (both *P* > 0.05). Also, Aβ1–40 and Aβ1–42 expression levels were lower in DSS-fed NLRP3 KO mice than DSS-fed WT mice (*P* < 0.0001, *P* < 0.01). Thus, colitis induced NLRP3-dependent Aβ accumulation in the cortex and hippocampus, possibly by suppressing glymphatic clearance, consistent with the observed neuropathology, neuroinflammation, and impaired spatial cognition.

### NLRP3 depletion decreased astrocytic activation induced by colitis

**Fig. 5:**
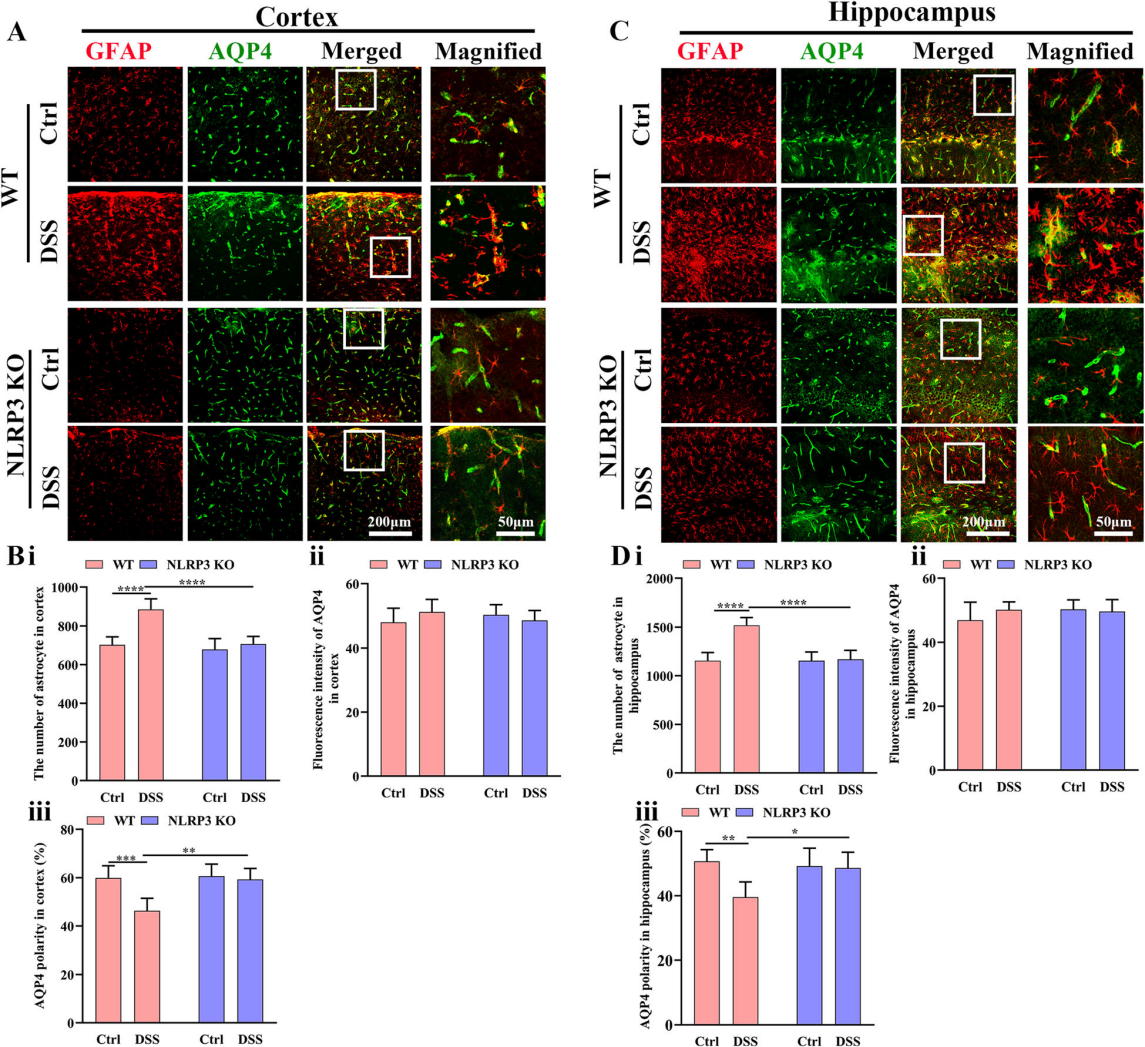
Colitis disrupted the polarity of astrocytic AQP4 distribution in WT but not NLRP3 KO mice. **A** Representative images of astrocytes and AQP4 expression in the cortex (Left 3 rows: 25× water immersion objective; right row: expanded images (3×) of regions in the white boxes). **B** Comparison of astrocyte number (i), AQP4 expression intensity (ii), and AQP4 polarity (iii) in the cortex among control WT, DSS-fed WT, NLRP3 KO, and DSS-fed NLRP3 KO mice. **C** Representative images of astrocytes and AQP4 expression in the hippocampus (Left three rows: 25× water immersion objective; right row: expanded images (3×) of regions in the white boxes). **D** Comparison of astrocyte number (i), AQP4 expression intensity (ii), and AQP4 polarity (iii) in the hippocampus among treatment groups. Each dataset is expressed as mean ± SD. *P ≤ 0.05; **P ≤ 0.01; ***P ≤ 0.001; ****P ≤ 0.0001. n = 6 mice

We then examined the effects of colitis and NLRP3 KO on astrocyte density and polarization of astrocytic AQP4 surface expression in the cortex and hippocampus as measures of astrocytic activation and function. In the cortex (Fig. 5A), the astrocyte density was higher in DSS-fed WT mice compared to control WT mice (*P* < 0.0001) but did not differ between DSS-fed and control NLRP3 KO mice (*P* > 0.05) and was significantly lower in DSS-fed NLRP3 KO mice than DSS-fed WT mice (*P* < 0.0001) (Fig. 5B i), suggesting that NLRP3 KO protected against neuroinflammation and reactive transformation of astrocytes. There were no significant pair-wise differences in AQP4 immunoexpression intensity among the four treatment groups (all *P* > 0.05) (Fig. 5B ii). Alternatively, the AQP4 polarity was significantly lower in DSS-fed WT mice than in control WT mice (*P* < 0.001). Conversely, there was no difference in polarity between DSS-fed and control NLRP3 KO mice (*P* > 0.05). Polarity was significantly higher in DSS-fed NLRP3 KO mice compared to DSS-fed WT mice (*P* < 0.001) (Fig. 5B iii).

In the hippocampus as well (Fig. 5C and D), astrocyte density was significantly higher in DSS-fed WT mice compared to control WT mice (*P* < 0.0001), but not significantly different between DSS-fed and control NLRP3 KO mice (*P* > 0.05). The density of hippocampal astrocytes was significantly lower in DSS-fed NLRP3 KO mice than DSS-fed WT mice (*P* < 0.0001) (Fig. 5D i). There were no pair-wise differences in hippocampal AQP4 expression among the four groups (all *P* > 0.05) (Fig. 5D ii). The polarity of AQP4 in the hippocampus was also significantly lower in DSS-fed WT mice than control WT mice (*P* < 0.001) but did not differ between DSS-fed and control NLRP3 KO mice (*P* > 0.05) and was significantly greater in DSS-fed NLRP3 KO mice than DSS-fed WT mice (*P* < 0.05) (Fig. 5D iii). These findings suggest that DSS induces neuroinflammation and impairs astrocytic function through enhanced NLRP3 inflammasome activity.

### NLRP3 depletion decreased the elevation in A1-like astrocyte numbers induced by colitis

**Fig. 6:**
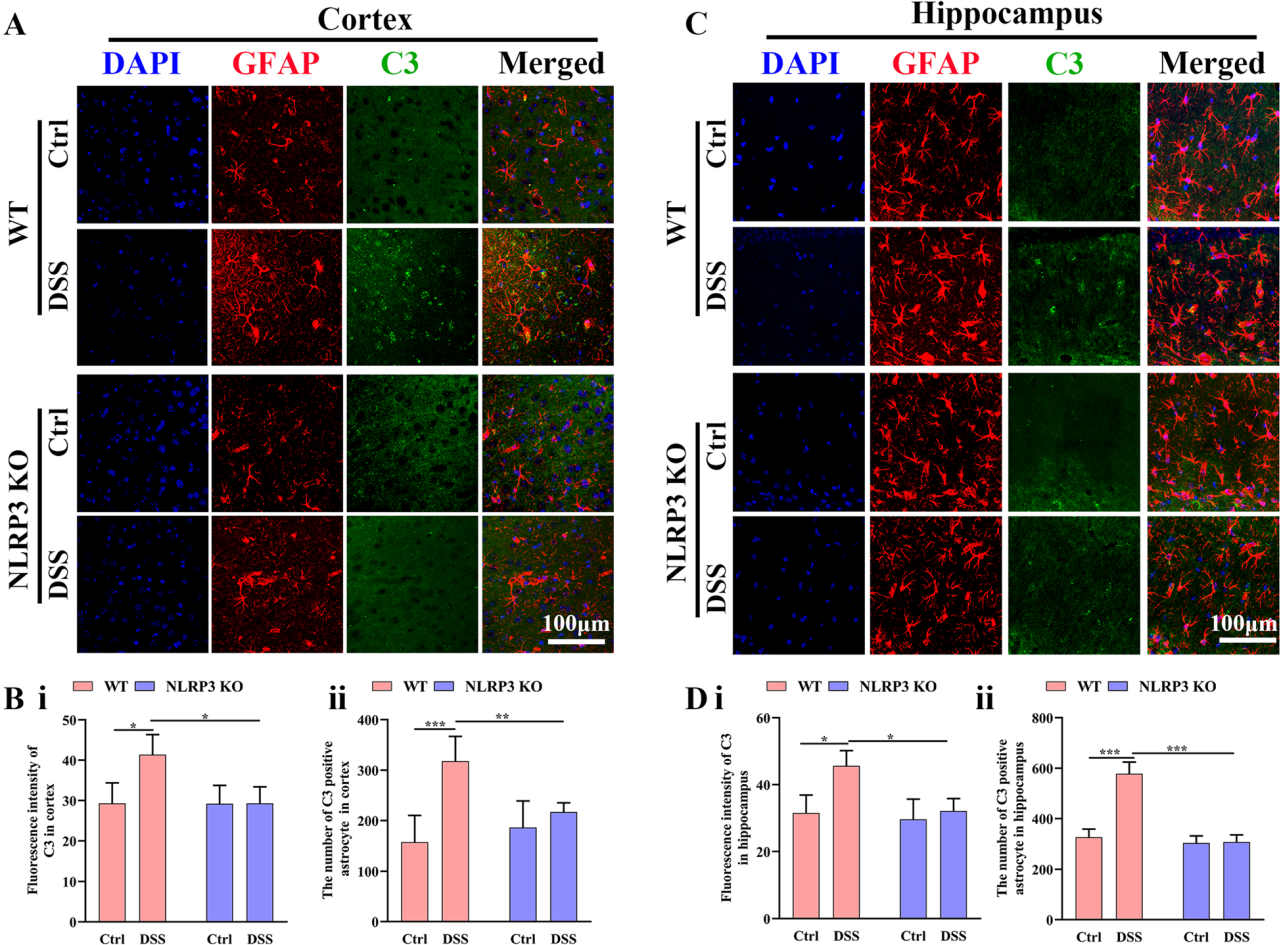
Colitis promoted transformation to the A1-like astrocyte phenotype in WT mice but not NLRP3 KO mice. **A** Representative images of C3 immunoexpression and astrocytes in the cortex (63× oil immersion objective). **B** Comparison of C3 immunofluorescence intensity (i) and C3-positive (A1-like) astrocyte number (ii) in the cortex among control WT, DSS-fed WT, NLRP3 KO, and DSS-fed NLRP3 KO mice. **C** Representative images of C3 immunoexpression and astrocytes in the hippocampus (63× oil immersion objective). **D** Comparison of C3 immunofluorescence intensity (i) and C3-positive (A1-like) astrocyte number (ii) in the hippocampus among treatment groups. Each dataset is expressed as mean ± SD. *P ≤ 0.05; **P ≤ 0.01; ***P ≤ 0.001; ****P ≤ 0.0001. n = 6 mice

We investigated the effect of DSS administration on astrocyte phenotype by GAFP and C3 immunofluorescence staining (Fig. 6). Administration of DSS significantly increased cortical C3 expression in WT mice (*P* < 0.05) but not NLRP3 KO mice (*P* > 0.05), and cortical C3 expression was significantly lower in DSS-fed NLRP3 KO mice than DSS-fed WT mice (*P* < 0.05) (Fig. 6 A and B i). Similarly, administration of DSS increased C3-positive astrocyte numbers in the cortex of WT mice (*P* < 0.001) but not NLRP3 KO mice (*P* > 0.05), and C3-positive astrocyte number was significantly lower in the cortex of DSS-fed NLRP3 KO mice compared to DSS-fed WT mice (*P* < 0.01) (Fig. 6A and B ii). In the hippocampus as well, C3 expression was significantly higher in DSS-fed WT than control WT mice (*P* < 0.05) but did not differ between DSS-fed and control NLRP3 KO mice (*P* > 0.05) and was also significantly lower in DSS-fed NLRP3 KO mice than DSS-fed WT mice (*P* < 0.05) (Fig. 6C and D i). Consistent with C3 expression levels, the number of C3-positive astrocytes in the hippocampus was significantly higher in DSS-fed mice than control WT mice (*P* < 0.001) but did not differ between DSS-fed and control NLRP3 KO mice (*P* > 0.05). The number of C3-positive hippocampal astrocytes was also significantly lower in DSS-fed NLRP3 KO mice than DSS-fed WT mice (*P* < 0.001) (Fig. 6C and D ii). These results indicated that DSS administration promoted the A1-like transformation of astrocytes and that this effect was dependent on NLRP3 activation.

### NLRP3 depletion rescued the impairment in glymphatic clearance induced by colitis

**Fig. 7:**
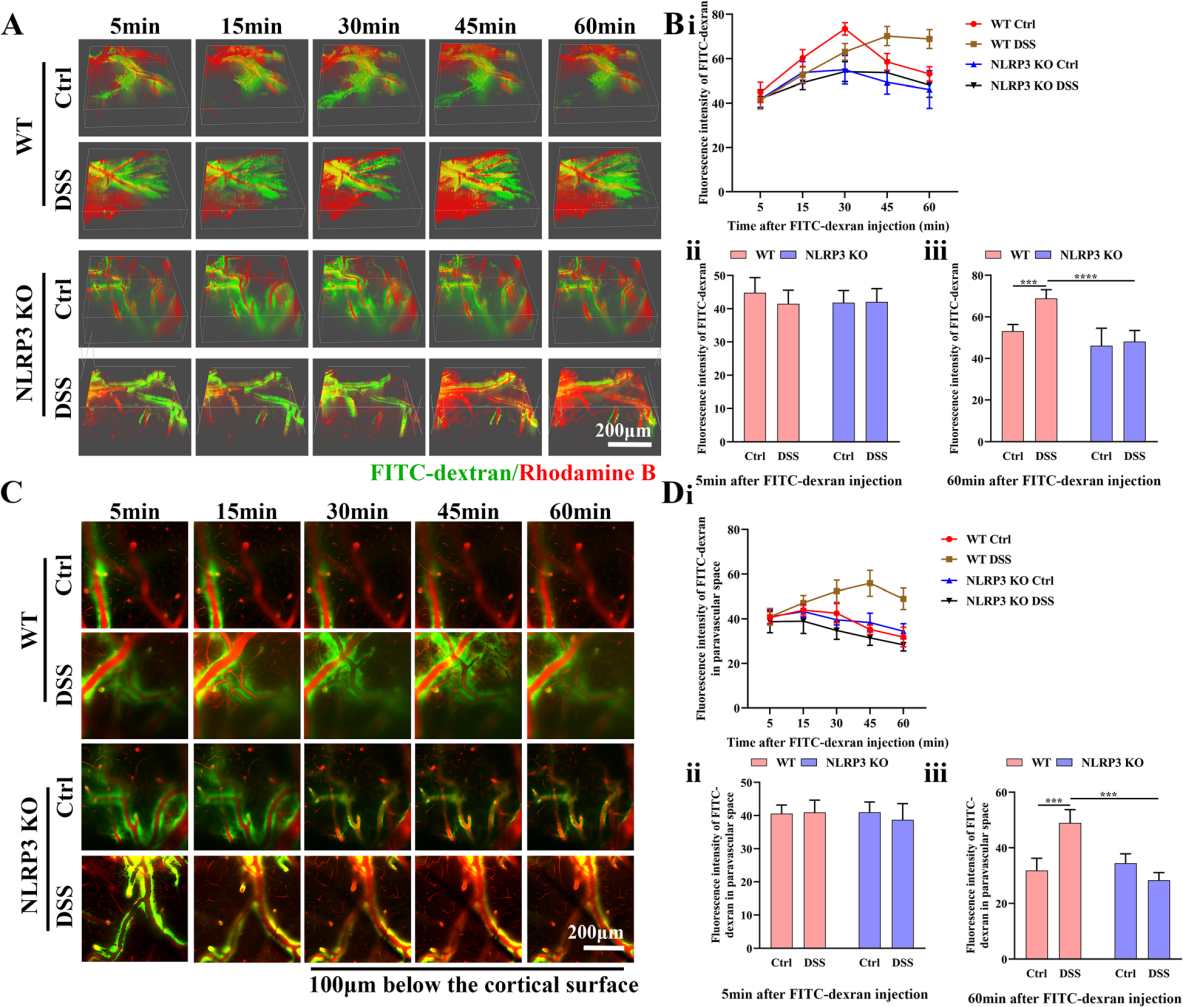
Colitis impaired glymphatic clearance in WT mice but not NLRP3 KO mice. **A** Representative three-dimensional images at 5, 15, 30, 45, and 60 min after infusion of FITC-dextran into the cisterna magna (25× water immersion objective). **B** Linear (i) and histogram (ii and iii) analyses of overall FITC-dextran intensity at different time points after infusion among control WT, DSS-fed WT, control NLRP3 KO mice, and DSS-fed NLRP3 KO mice. **C** Representative two-dimensional images 100 μm below the cortical surface at 5, 15, 30, 45, and 60 min after infusion of FITC-dextran into the cisterna magna (25× water immersion objective). **D** Linear (i) and histogram (ii and iii) analyses of FITC-dextran intensity in the paravascular space at different time points among control WT, DSS-fed WT, control NLRP3 KO, and DSS-fed NLRP3 KO mice. Each dataset is expressed as mean ± SD. *P ≤ 0.05; **P ≤ 0.01; ***P ≤ 0.001; ****P ≤ 0.0001. n = 6 mice

To directly examine the effect of colitis on glymphatic function, we measured FITC-dextran clearance in vivo using two-photon imaging (Fig. 7). Following intracisternal injection, the FITC tracer moved along the cerebral vasculature and entered the cortical parenchyma. Three-dimensional analysis revealed a progressive rise in FITC fluorescence intensity for 30 min before decreasing in WT control and both NLRP3 groups, while the tracer signal continued to increase for 60 min in DSS-fed WT mice, suggesting impaired glymphatic clearance (Fig. 7A and B i). There were no significant pair-wise differences among groups (all *P* > 0.05) at 5 min after tracer injection (Fig. 7A and B ii). At 60 min after tracer injection, however, tracer fluorescence intensity was significantly greater in DSS-fed than control WT mice (*P* < 0.001) but did not differ between NLRP3 KO groups (*P* > 0.05), and was significantly lower in DSS-fed NLRP3 KO mice than DSS-fed WT mice (*P* < 0.0001) (Fig. 7A and B iii).

To examine the efficiency of interstitial fluid flow, we then analyzed FITC-dextran movement in the paravascular space at 100 μm below the cortical surface (Fig. 7C and D). In DSS-fed WT mice, FITC signal intensity kept increasing from 5 to 45 min post-injection, and then gradually decreased, while all other groups demonstrated decreases in signal intensity starting 5 min following injection (Fig. 7C and D i). Specifically, at 5 min following injection, there were no significant differences in FITC intensity among the four groups at 5 min post-injection (all *P* > 0.05) (Fig. 7C and D ii). At 60 min following FITC-dextran injection, however, FITC intensity was significantly higher in DSS-fed WT mice than control WT mice (*P* < 0.001) but did not differ between NLRP3 KO groups (*P* > 0.05) and was significantly lower in DSS-fed NLRP3 KO mice compared to DSS-fed WT mice (*P* < 0.001) (Fig. 7C and D iii). Collectively, these results indicated that DSS-induced colitis and neural NLRP3 inflammasome activity impaired glymphatic clearance, while NLRP3 inflammasome suppression mitigated this effect.

### NLRP3 depletion inhibited meningeal accumulation of gut-derived T cells

**Fig. 8:**
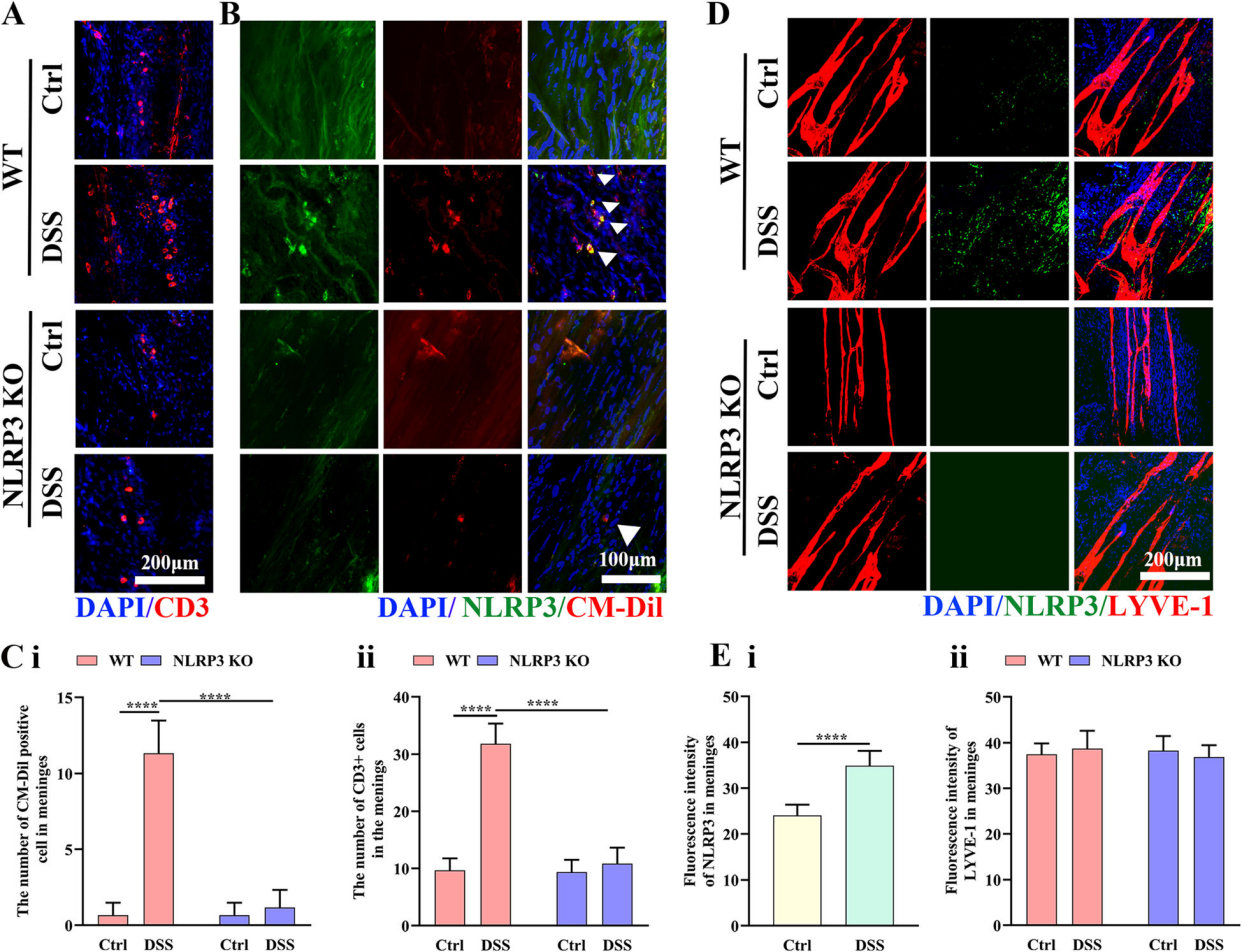
Colitis increased NLRP3 inflammasome expression and accumulation of gut-derived cells in the meninges of WT mice but not NLRP3 KO mice. **A** Representative images of CD3 immunoexpression in the meninges (25× water immersion objective). **B** Representative images of CM-Dil-positive (gut-derived) cells and NLRP3 expression in the meninges (25× water immersion objective, magnified 3×). **C** Comparison of CD3-positive cell number (i) and CM-Dil-positive cell number (ii) in the meninges of WT and NLRP3 KO mice. **D** Representative images of NLRP3 inflammasome and LYVE-1 immunoexpression in the meninges (25× water immersion objective). **E** Comparison of NLRP3 inflammasome (i) and LYVE1 intensities (ii) in the meninges of WT and NLRP3 KO mice. Each dataset is expressed as mean ± SD. *P ≤ 0.05; **P ≤ 0.01; ***P ≤ 0.001; ****P ≤ 0.0001. n = 6 mice

A previous study reported that the neuroinflammation associated with colitis was enhanced by migration of activated γδ T cells from the gut to the meninges [33], so we examined if the differences in neuroinflammation and functional deficits among groups reflected meningeal gut-derived T cell accumulation (Fig. 8). There were a significantly greater number of CD3-positive cells in the mLVs of DSS-fed WT mice than control WT mice (*P* < 0.0001) but not in the mLVs of DSS-fed NLRP3 KO mice compared to control NLRP3 KO mice (*P* > 0.05) (Fig. 8A and C i). In addition, there were a significantly greater number of CM-Dil-positive cells in the mLVs of DSS-fed WT mice than control WT mice (*P* < 0.0001) but not in the mLVs of DSS-fed NLRP3 KO mice compared to control NLRP3 KO mice (*P* > 0.05) (Fig. 8B and C ii). Moreover, CM-Dil-positive cell number was significantly lower in the mLVs of DSS-fed NLRP3 KO mice compared to DSS-fed WT mice (*P* < 0.0001) (Fig. 8B and C ii). It was previously confirmed that CM-Dil microinjection into PPs only labels cells located within PPs without systemic cell labeling (i.e., in blood or spleen) [27]. Therefore, these findings suggest that recruitment of gut-derived lymphocytes to the meninges is dependent on NLRP3 inflammasome activity. There was no obvious NLRP3 inflammasome activity in the meninges of NLRP3 KO mice, while DDS feeding significantly enhanced NLRP3 inflammasome activity in WT mice (t = 6.68, *P* < 0.0001) (Fig. 8D and E i), but for expressions of the lymphatic endothelial cell marker, there was no difference in LYVE-1 intensity among groups (all *P* > 0.05) (Fig. 8D and E ii).

## Discussion

There is compelling evidence that the gut–brain axis (GBA) regulates the progression of neurodegenerative diseases, including AD [34, 35], and that intestinal lesions even decades before AD diagnosis may accelerate the underlying neuropathological processes [2]. Consistent with this notion that intestinal lesions can exacerbate early AD-like pathology, intestinal inflammation induced by DSS disrupted glymphatic clearance, increased Aβ deposition, triggered neuroinflammation, impaired spatial cognition, and induced anxiety-like behavior among aging mice. Furthermore, these pathological effects were dependent on the NLRP3 inflammasome as all were suppressed by NLRP3 *knockout*, underscoring the therapeutic potential NLRP3 inflammasome blockade for early treatment of AD.

Individuals with IBD are reported to be at increased risk of developing anxiety or depression [36], and patients with a history of anxiety or depression are at increased risk of dementia and cognitive impairment [37–39]. Consistent with a mediating role for anxiety in colitis-associated cognitive impairment, anxiety-like behavior in the open field was accompanied by impaired spatial memory during the MWM probe trial and with reduced novel object recognition. Normal animals exhibit a preference to explore novel objects over familiar objects, so reduced attention or interaction with novel objects is a sign of recognition memory dysfunction [25]. In contrast, DSS-induced colitis did not affect the escape latencies during MWM training, consistent with a previous study reporting that colitis impairs spatial memory without influencing spatial learning [40].

Microglial activation by the NLRP3 inflammasome may be a critical contributor to increased Aβ deposition. First, microglia are the resident macrophages and primary immune cells in the central nervous system responsible for the phagocytosis and clearance of Aβ [41] and NLRP3 activation was found to promote pro-inflammatory responses of microglia and dampen Aβ clearance [42–44]. Moreover, astrocytes are also responsible for Aβ clearance [45], and microglial activation induces A1-like astrocyte transition, resulting in a functionally deficient phenotype [10, 11]. Consistent with these findings, DSS-induced colitis increased microglial activation and A1-like astrocyte numbers, and these responses were inhibited by NLRP3 depletion.

Dysfunction of glymphatic clearance has been shown to enhance Aβ deposition [46], and indeed, DSS-fed WT mice showed greater Aβ accumulation concomitant with reduced glymphatic clearance of a dextran tracer as well as multiple additional signs of glymphatic impairment such as loss of AQP4 polarity. As a lymphatic-like system in the brain, the glymphatic pathway drains protein wastes to the cervical lymphatics both in humans and mice [8, 47]. Surface expression of AQP4 must be polarized to astrocytic endfeet abutting cerebral vessels for efficient glymphatic clearance [10, 48], and the reactive astrocytes from DSS-fed WT mice demonstrated reduced AQP4 polarity. The pro-inflammatory cytokine IL-1β is considered a key mediator of NLRP3-induced glymphatic dysfunction. First, NLRP3 triggers the maturation of IL-1β, which binds to cognate receptors on astrocytes leading to astrogliosis [49] and loss of AQP4 polarity, while depletion of NLRP3 decreases mature IL-1β production and improves AQP4 polarity. Mature IL-1β binding to IL-1 receptors was reported to upregulate the expression of pro-IL-1β [50]. Moreover, reactive A1-like astrocytes in the aged brain produce an exaggerated response to IL-1β [11]. In addition to Aβ, glymphatic dysfunction may also lead to enhanced formation of neurofibrillary tangles. Tau protein can be transferred between cells through the fluid in extracellular space, so glymphatic dysfunction may exacerbate neuron-to-neuron propagation [8], a process that will be explored in our future studies.

Accumulation of gut-derived T cells in mLVs is another potential contributor to age-related neuropathology and cognitive decline [33]. Meningeal LVs alter the accessibility of immune neuromodulators to the brain parenchyma, thereby potentially exacerbating inflammation [51]. In addition, CD4 + T cells in the meninges were reported to enter the CSF [52], induce microglial activation, and enhance local pro-inflammatory cytokine production [33, 53, 54]. Using in vivo CM-Dil cell tracing, we found that CD4+T cells in the paracolic lymph nodes migrated to the meninges. Furthermore, brain cytokines have been demonstrated to promote T cell infiltration [19]. The reduced neuropathology observed in NLRP3 KO mice following DSS-induced colitis may have resulted from lower mature IL-1β production and ensuing infiltration of fewer gut-derived T cells.

Dysfunction of mLVs due to DSS treatment may also contribute to Aβ plaque accumulation [55]. However, LYVE-1 staining indicated that meningeal lymphatic endothelial cells were unaffected by colitis or NLRP3 depletion. Indeed, mLVs express a unique transcriptional signature, and meningeal lymphatic endothelial cells do not undergo expansion during inflammation [55, 56]. There is another limitation in this study, which will be explored in the future. We cannot exclude the possibility that the protection conferred by NLRP3 depletion was due to attenuation of experimental colitis [57, 58] as depletion also decreased the histological colitis score.

## Conclusion

We demonstrate that intestinal inflammation can trigger neuroinflammation and decreased glymphatic clearance efficacy in aging mice, resulting in increased Aβ deposition and ultimately in neuronal death and cognitive impairment. We further demonstrate that these effects are likely mediated in part by migration of gut-derived CD4+ T cells and activation of the NLRP3 inflammasome. These mechanisms linking gut to brain inflammation are potential therapeutic targets for the treatment of neurodegenerative diseases such as AD.

## Supplementary Information

**Additional file 1:.** Supplementary figure 1. NLRP3 inflammasome upregulation induced by colitis was confined to astrocytes and microglia, but did not occur in neurons. A. Co-immunofluorescence staining of NLRP3 and Iba 1. B. Co-immunofluorescence staining of NLRP3 and GFAP. C. Co-immunofluorescence staining of NLRP3 and Neun.

**Additional file 2:.** Supplementary figure 2. Colitis increased brain Aβ accumulation as detected by western blots in WT mice but not NLRP3 KO mice. A. Chemiluminescence imaging of western blots showing that DSS administration did not affect APP expression in either control WT or NLRP3 KO mice, while DSS feeding increased Aβ 40 expression in control WT mice but not NLRP3 KO mice. B. Comparisons of APP/β-tubulin (i) and Aβ 40/β-tubulin (ii) ratios among control WT, DSS-fed WT, control NLRP3 KO, and DSS-fed NLRP3 KO mice. C. Chemiluminescence imaging of western blots showing that DSS administration increased Aβ 42 expression in WT mice but not NLRP3 KO mice. D. Comparisons of Aβ 42/β-tubulin ratio among control WT, DSS-fed WT, control NLRP3 KO, and DSS-fed NLRP3 KO mice. Each dataset is expressed as mean ± SD. *P ≤ 0.05; **P ≤ 0.01; ***P ≤ 0.001; ****P ≤ 0.0001. n = 3 mice.

## Data Availability

All data generated or analyzed during this study are included in this published article (and its supplementary information files).

## Abbreviations

IBDs: Inflammatory bowel diseases
NLRP3: NACHT-LRR and pyrin (PYD) domain-containing protein 3
DSS: Dextran sodium sulfate
Aβ: Amyloid-beta
AD: Alzheimer’s disease
mLVs: Meningeal lymphatic vessels
KO: *Knockout*
IL-1β: Interleukin-1β
ASC: Apoptosis-associated speck
MAP2: Microtubule-associated protein 2
ANOVA: Analysis of variance
AQP4: Aquaporin-4
GAFP: Glial fibrillary acidic protein
Iba1: Ionized calcium-binding adapter molecule 1
FITC: Fluorescein isothiocyanate
GBA: Gut–brain axis
CSF: Cerebrospinal fluid
ISF: Interstitial fluid
