# Correction to: Chronic colitis exacerbates NLRP3-dependent neuroinflammation and cognitive impairment in middle-aged brain

**DOI:** 10.1186/s12974-021-02347-0

**Published:** 2022-01-15

**Authors:** Xiao-fei He, Li-li Li, Wen-biao Xian, Ming-yue Li, Li-ying Zhang, Jing-hui Xu, Zhong Pei, Hai-qing Zheng, Xi-quan Hu

**Affiliations:** 1 Department of Rehabilitation Medicine, The Third Affiliated Hospital, Sun Yat-sen University, 600 Tianhe Road, Guangzhou, 510630 Guangdong China; 2 Department of Neurology, National Key Clinical Department and Key Discipline of Neurology, Guangdong Key Laboratory for Diagnosis and Treatment of Major Neurological Diseases, The First Affiliated Hospital, Sun Yat-sen University, Guangzhou, 510080 Guangdong China

## Correction to: Journal of Neuroinflammation (2021) 18:153 10.1186/s12974-021-02199-8

The original version of the article [1] unfortunately contained a mistake IN Figs. 2 and 8.

**Fig. 2:**
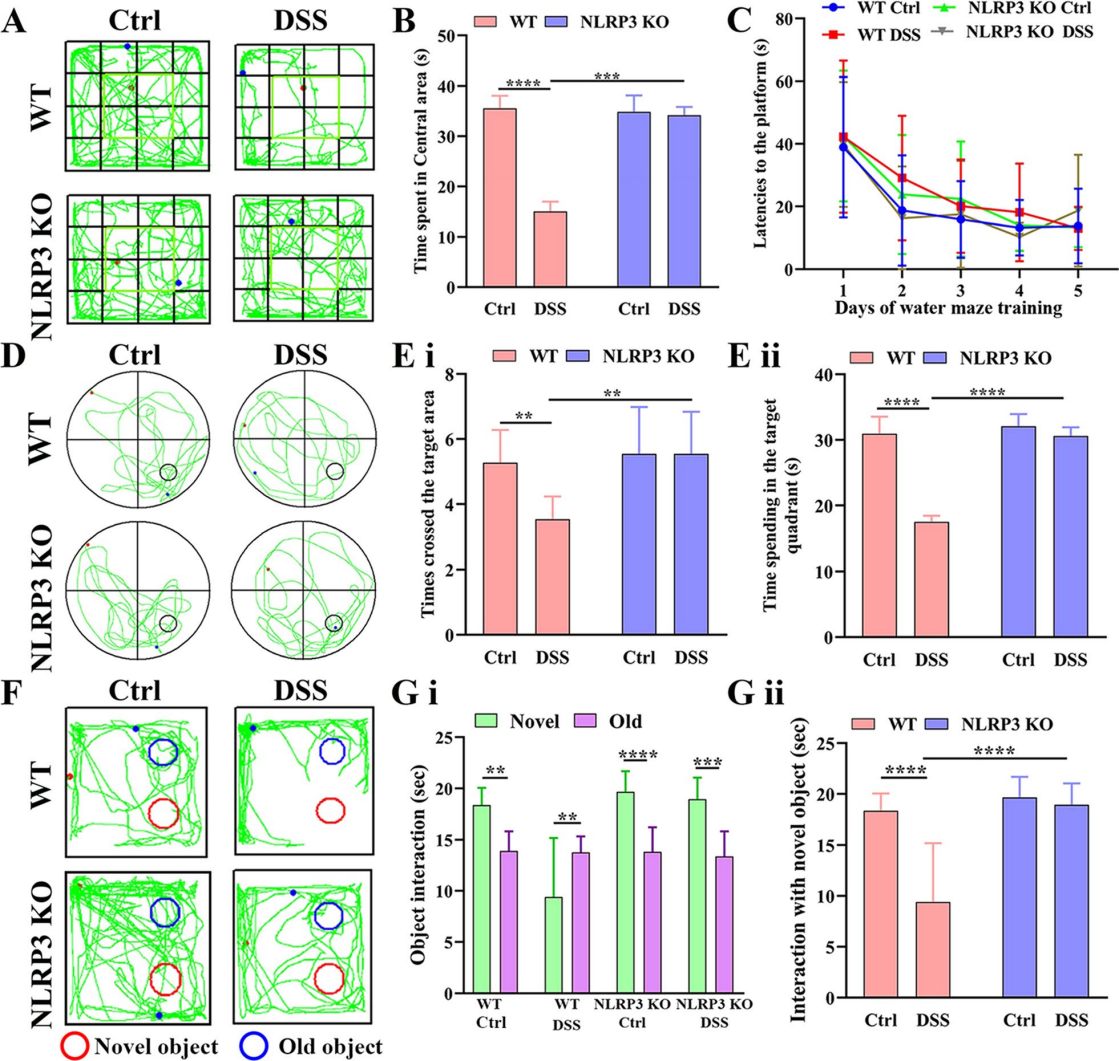
Induction of colitis by addition of DSS to drinking water (1% vol/vol) for 4 weeks altered spontaneous motor behavior and impaired spatial and recognition memory in aged (16-month-old) wild-type (WT) mice but not age-matched NLRP3 KO mice. **A** Representative movement tracks in the open field test showing less time spent in the central area by DSS-fed WT mice compared to untreated control (Ctrl) WT mice but no difference between DSS-fed and control NLRP3 KO mice. **B** Comparison of time spent in the central area of the open field by all 4 treatment groups. **C** Comparison of latency to the platform during the 5 days of Morris water maze training. **D** Representative swim paths during the probe trial for spatial memory showing that DSS-fed WT mice made fewer crossings over the former platform location and spent less swim time in the target quadrant than control WT mice, indicating spatial memory impairment, while these values did not differ between DSS-fed and control NLRP3 KO mice. **E** Comparison of times crossing the former target area (i) and time spent in the target quadrant in the probe trial (ii). **F** Representative movement tracks in the novel object test showing that DSS-fed WT mice spent equal time contacting the familiar and novel objects, while mice in other treatment groups spent more time in contact with the novel object. **G** Comparison of the time spent in contact with the novel and familiar objects by all 4 treatment groups (i) and comparison of the time spent in contact with the novel object among the four groups (ii). Each dataset is expressed as mean ± SD. *P ≤ 0.05; **P ≤ 0.01; ***P ≤ 0.001; ****P ≤ 0.0001. *n* = 12 mice

**Fig. 8:**
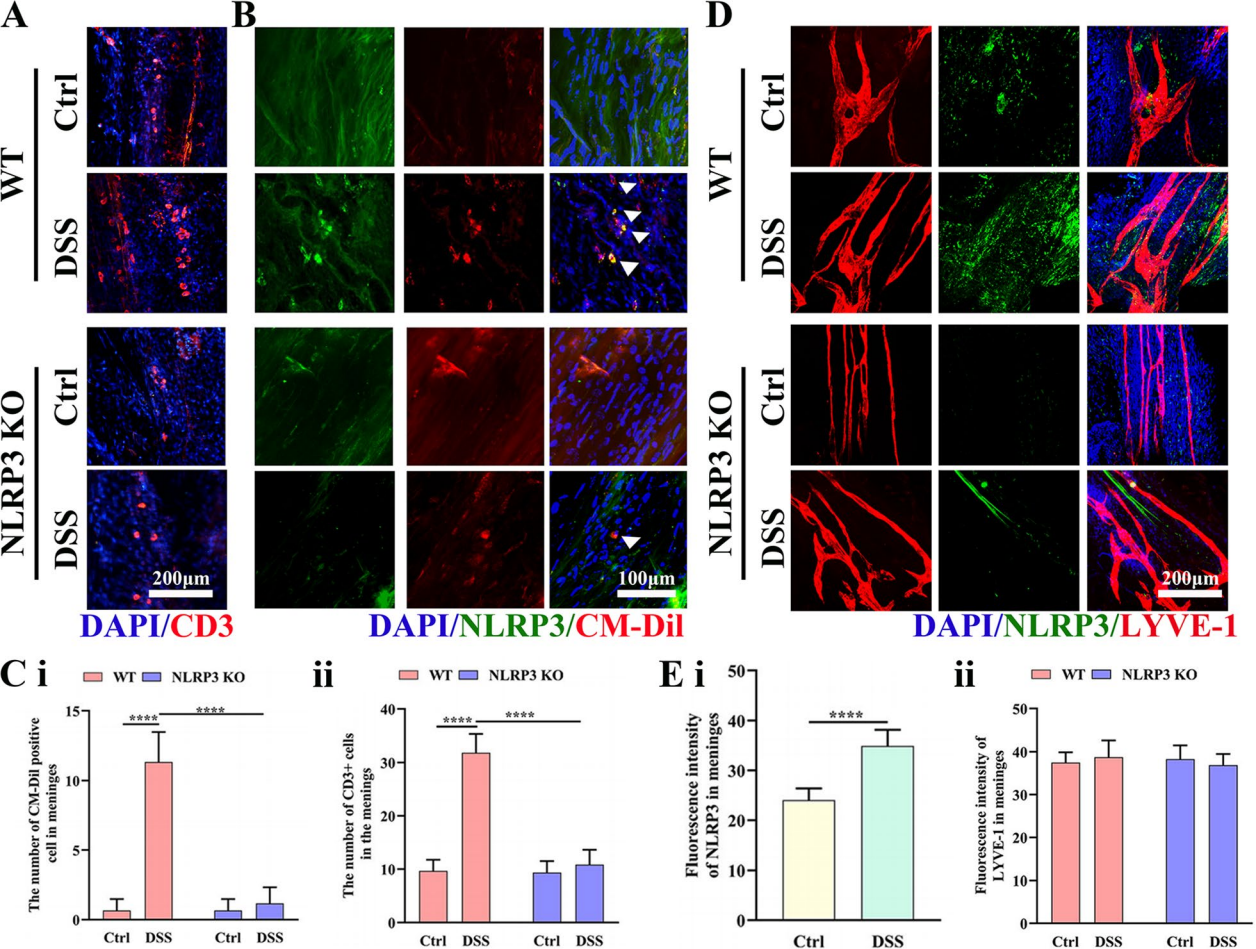
Colitis increased NLRP3 inflammasome expression and accumulation of gut-derived cells in the meninges of WT mice but not NLRP3 KO mice. **A** Representative images of CD3 immunoexpression in the meninges (25 × water immersion objective). **B** Representative images of CM-Dilpositive (gut-derived) cells and NLRP3 expression in the meninges (25 × water immersion objective, magnified 3 ×). **C** Comparison of CD3-positive cell number (i) and CM-Dil-positive cell number (ii) in the meninges of WT and NLRP3 KO mice. **D** Representative images of NLRP3 inflammasome and LYVE-1 immunoexpression in the meninges (25 × water immersion objective). **E** Comparison of NLRP3 inflammasome (i) and LYVE1 intensities (ii) in the meninges of WT and NLRP3 KO mice. Each dataset is expressed as mean ± SD. *P ≤ 0.05; **P ≤ 0.01; ***P ≤ 0.001; ****P ≤ 0.0001. *n* = 6 mice

The corresponding author for the manuscript titled by “Chronic colitis exacerbates NLRP3-dependent neuroinflammation and cognitive impairment in middle-aged brain” that was published in your journal, Journal of Neuroinflammation, volume 18, Article number: 153 (2021). After publication of the article, we recently identified after re-reviewing this manuscript that the images from the “NLRP3 KO control” and “NLRP3 KO DSS” in Fig. 2. F showed overlap due to mistaken figure production, and the “LYVE-1” images of “WT Control” and “NLRP3 KO DSS” showed overlap with the “LYVE-1” images of “WT DSS” in Fig. 8 due to mistakenly capturing the image from the “LYVE-1” images of “WT DSS” repetitively, leading to the duplication of the WT DSS images for “WT Control” and “ NLRP3 DSS”. The serious mistake was caused by unintentionally and carelessly mistaking the “WT Control” or “NLRP3 DSS” instead of “WT DSS” during image capturing. In fact, the careless mistakes do not affect the findings or conclusions of the article. To effectively modify this issue, and prevent further misunderstanding with regard to this mistake accordingly, we would appreciate it if it would be possible to request erratum for this mistake and we have attached the corrected figures. If permitted, we would like to re-perform the relevant experiments to confirm the accurate and repeatability regarding the effect of NLRP3 inflammasome on the of colits-induced neuroinflammation.

Really apologize for any inconvenience that our careless mistake may have caused.
