# Supplementary figures and images for: Chronic colitis exacerbates NLRP3-dependent neuroinflammation and cognitive impairment in middle-aged brain
Source: J Neuroinflammation. 2021 Jul 6;18:153. doi: 10.1186/s12974-021-02199-8 (PMC8262017; doi:10.1186/s12974-021-02199-8)

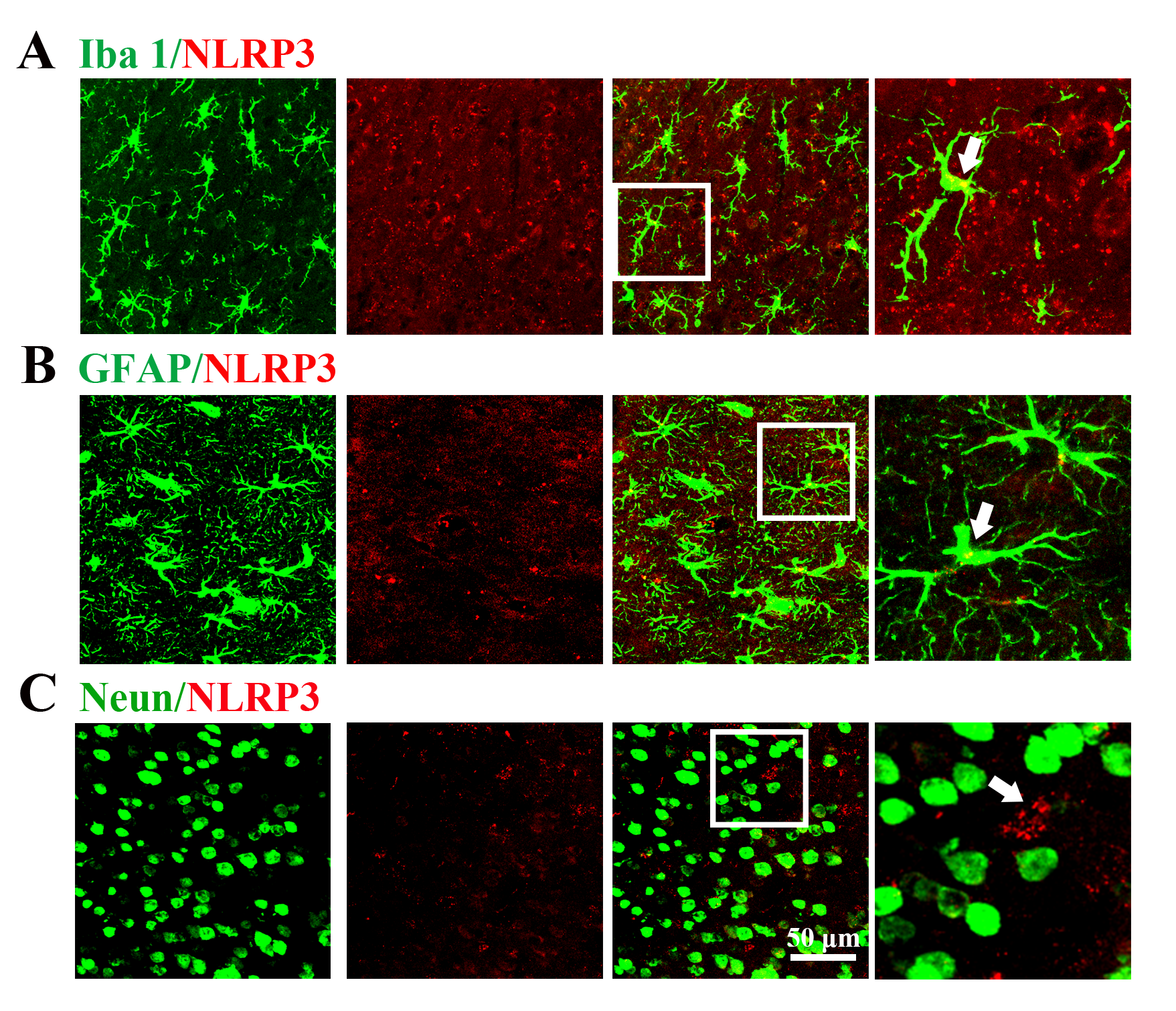

Supplement: Supplementary file 1 — Additional file 1:. Supplementary figure 1. NLRP3 inflammasome upregulation induced by colitis was confined to astrocytes and microglia, but did not occur in neurons. A. Co-immunofluorescence staining of NLRP3 and Iba 1. B. Co-immunofluorescence staining of NLRP3 and GFAP. C. Co-immunofluorescence staining of NLRP3 and Neun. [file 12974_2021_2199_MOESM1_ESM.tif]

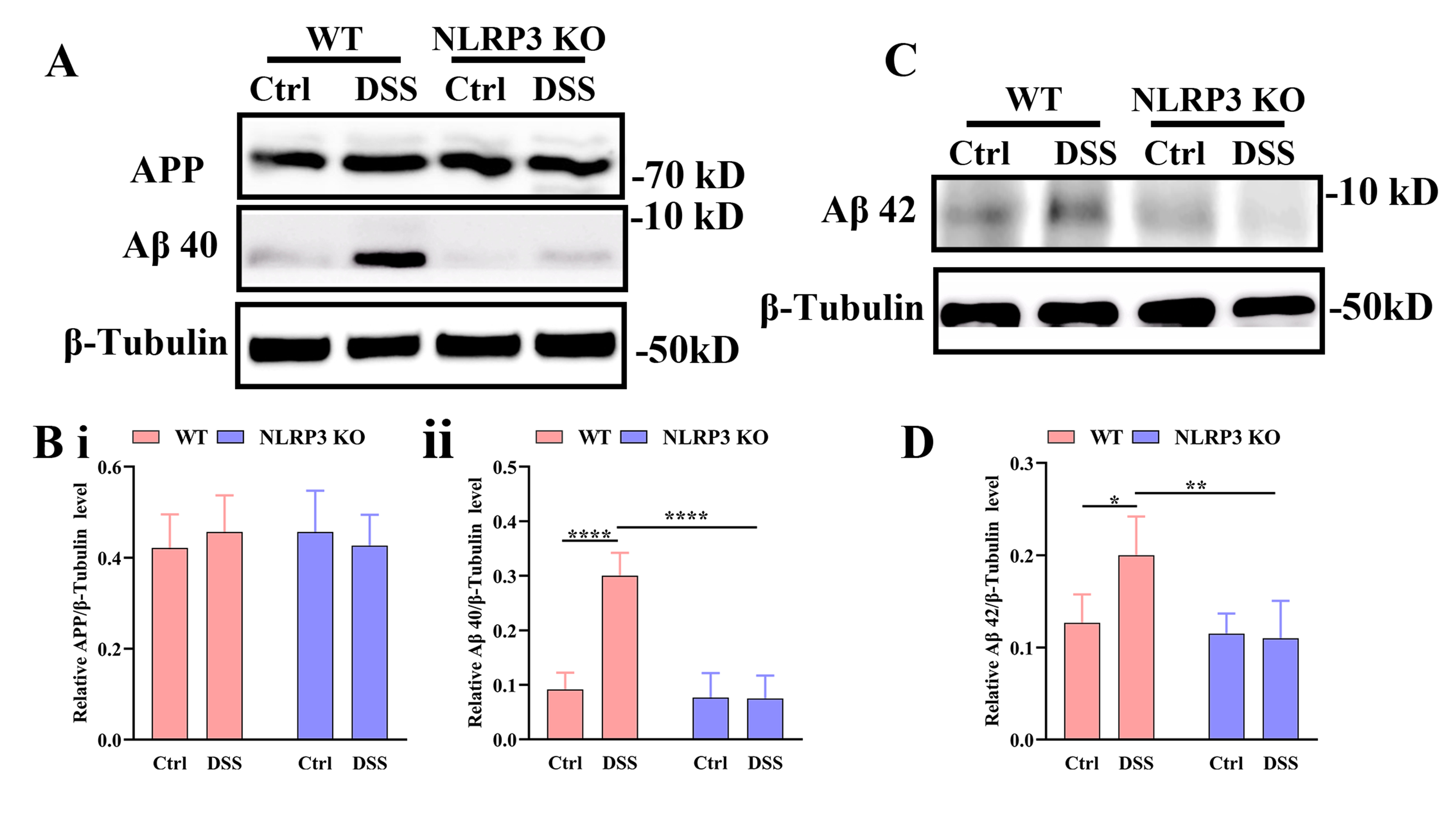

Supplement: Supplementary file 2 — Additional file 2:. Supplementary figure 2. Colitis increased brain Aβ accumulation as detected by western blots in WT mice but not NLRP3 KO mice. A. Chemiluminescence imaging of western blots showing that DSS administration did not affect APP expression in either control WT or NLRP3 KO mice, while DSS feeding increased Aβ 40 expression in control WT mice but not NLRP3 KO mice. B. Comparisons of APP/β-tubulin (i) and Aβ 40/β-tubulin (ii) ratios among control WT, DSS-fed WT, control NLRP3 KO, and DSS-fed NLRP3 KO mice. C. Chemiluminescence imaging of western blots showing that DSS administration increased Aβ 42 expression in WT mice but not NLRP3 KO mice. D. Comparisons of Aβ 42/β-tubulin ratio among control WT, DSS-fed WT, control NLRP3 KO, and DSS-fed NLRP3 KO mice. Each dataset is expressed as mean ± SD. *P ≤ 0.05; **P ≤ 0.01; ***P ≤ 0.001; ****P ≤ 0.0001. n = 3 mice. [file 12974_2021_2199_MOESM2_ESM.tif]
